# Supplementary material for: Creative connections: the neural correlates of semantic relatedness are associated with creativity
Source: Commun Biol. 2024 Jul 4;7:810. doi: 10.1038/s42003-024-06493-y (PMC11222432; doi:10.1038/s42003-024-06493-y)
Supplement: Supplementary file 2 — Supplementary Information [file 42003_2024_6493_MOESM2_ESM.pdf]

SUPPLEMENTARY INFORMATION

Creative connections: the neural correlates of semantic relatedness are associated with creativity

Caroline Herault[1]*, Marcela Ovando-Tellez[1]*, Izabela Lebuda[2, 3], Yoed N. Kenett[4], Benoit Beranger[5], Mathias Benedek[2] & Emmanuelle Volle[1]

[1] Sorbonne University, FrontLab at Paris Brain Institute (ICM), INSERM, CNRS, 75013, Paris, France

[2] Institute of Psychology, University of Graz, Graz, Austria

[3] Institute of Psychology, University of Wroclaw, Wroclaw, Poland

[4] The Faculty of Data and Decision Sciences, Technion – Israel Institute of Technology, Haifa 3200003 Israel.

[5] Sorbonne University, CENIR at Paris Brain Institute (ICM), INSERM, CNRS, 75013, Paris, France

*** These authors contributed equally**

Corresponding authors:

Caroline Herault: caroline.herault38@gmail.com

Emmanuelle Volle: emmavolle@gmail.com

Paris Brain Institute – GHPS

47 bd de l'hopital

75013 Paris

PART 1- RJT TASK

Supplementary Methods 1. Theoretical semantic distance between the RJT words

Based on the findings that steps in a semantic memory network reliably reflect a semantic distance, Bernard et al. [1] employed computational methods to build a French semantic network using French verbal association norms (http://dictaverf.nsu.ru/pages/dict_sanfn.php?lang=fr). The authors created a French semantic memory network composed of 1,081 nodes (French words) connected by links whose weights represent the semantic association estimated from the association norms. The authors created hierarchical tree structures to select 35 words (for experimental reasons) that optimized the proportion of word pairs separated by 1, 2, 3, 4, and 5 or more steps when considering all possible word pair combinations. Adapting the method from [2], Bernard et al. [1] used a relatedness judgment task (RJT) in which participants are presented with all possible pairs of these 35 words and asked to rate their semantic relatedness. The results from this study showed that higher creativity in terms of real-life creative abilities and achievements correlated with higher average relatedness judgments especially in distant word-pairs (4 to 6 steps). These results indicate the close link between creativity and seeing things as related, which was also demonstrated by [2] and [3]. Hence, the current study used the same material and RJT task as in [1] and [3].

Of note, at the difference with the present study, this previous work used the relatedness judgments during the RJT to estimate individual-based semantic memory networks, where each word represents a node and the relatedness ratings given by the participant represent the links connecting these nodes. Converging results from these studies showed that higher creative ability (measured with divergent thinking tasks) [2] and more creative behavior in real-life [3] were associated with lower average shortest path lengths (ASPL) suggesting a more efficient semantic memory network structure in creative people (see also [4]). Ovando-Tellez et al. [3] additionally showed that higher creative achievements were related to a less segregated network (lower modularity) [3]. In addition, Ovando-Tellez et al. [3] used a connectome predictive modeling approach, and identified patterns of brain functional connectivity that predicted semantic memory network properties relevant to creativity. In contrast, here we explored the brain fMRI activity directly related to the RJT judgments.

Supplementary Table 1. Verbal material of the Relatedness Judgment task (RJT). *The 35 words used in the task are presented in the table, with the translation in English in brackets. Note that the translation does not take into account the polysemy that exists in French for some of these words.*

List of RJT words			
bouche [mouth]	existence [existence]	expression [expression]	mort [death]
nez [nose]	terme [term]	figure [shape]	espoir [hope]
condition [condition]	suite [continuation]	gros [fat]	bout [tip]
détail [detail]	parti [party]	lettre [letter]	ministre [minister]
expérience [experience]	fleur [flower]	face [face]	français [french]
discours [speech]	propos [statement]	front [forehead]	
calme [calm]	joue [cheek]	bruit [noise]	
mot [word]	épaule [shoulder]	dire [to say]	
dent [tooth]	cheveu [hair]	voix [voice]	
grand [large]	cou [neck]	langue [tongue]	

Supplementary Analysis 1. Combining trials with steps 5 and 6

We combined trials with 5 and 6 steps as the trials with 6 steps were only 24 in number. We considered that using only 24 trials (4% of the total number of trials) would not have enough power for the analysis of these trials independently. We ran a t-test to compare the two types of trials (step 5 and step 6): the result confirmed that there was no significant difference between these steps (t = -.424, *p*-value = .672).

Supplementary Figure 1. Distribution of within subject average ratings for each theoretical step, separating trials of 5 and 6 steps. Each boxplot represents the distribution of ratings for individual theoretical steps, with outliers represented by the symbol "+".

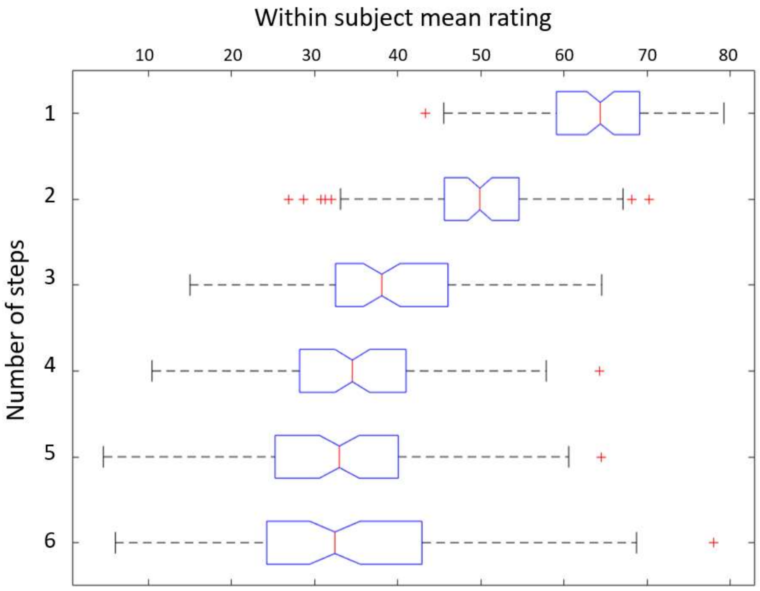

PART 2- CREATIVITY ASSESSMENT AND RJT RATINGS

Supplementary Table 2: Correlations between creativity scores and RTJ ratings, step-by-step. *The significant p-values (< .05) are displayed in bold font.*

		**Mean rating of Step 1**	**Mean rating of Step 2**	**Mean rating of Step 3**	**Mean rating of Step 4**	**Mean rating of Step 5 & 6**
C-Ach	R	.108	.236	.278	.286	.258
	p-value	*.302*	***.023***	***.007***	***.005***	***.013***
C-Act	R	.085	.208	.242	.253	.219
	p-value	*.418*	***.045***	***.019***	***.014***	***.036***
CAT	R	.170	.163	.0794	.043	.107
	p-value	*.103*	*.118*	*.449*	*.681*	*.306*
AUT fluency	R	-.146	-.133	-.131	-.113	-.133
	p-value	*.162*	*.203*	*.212*	*.282*	*.203*
AUT freq	R	-.174	-.246	-.194	-.237	-.315
	p-value	*.096*	***.017***	*.063*	***.022***	***.002***
AUT ext	R	.023	.072	-.001	.015	.091
	p-value	*.828*	*.491*	*.995*	*.885*	*.382*

PART 3- INDIVIDUAL VARIABILITY FOR RJT RATING

Supplementary Analysis 2: Behavioral analysis of individual variability for RJT rating

As it may affect the step-by-step fMRI parametric analysis of the rating, we evaluated whether there was an edge effect on the theoretical distance scale (steps) when analyzing the ratings at the individual level. The repeated measures ANOVA on the 5 distributions of ratings standard deviations depending on the step revealed significant differences between them (F = 36.9, $p < .05$, df = 4). Post-hoc analysis with a Bonferroni adjustment found significant differences in the rating standard deviation between all pairs of steps compared, besides between steps 1 and 3 (t = .29, p =.77) and step 4 and 5 (t = .54, p =.59) (Supplementary Table 3 and Supplementary Figure 2), suggesting that both edges of the scale (step 1 and step 5) have at as much variance with a middle scale step (respectively step 3 and step 4).

Supplementary Table 3. Pair-wise t-tests comparing the relatedness rating standard deviations for different theoretical distances (steps). *In bold font: p < .05*

Compared groups	*p*-value	Bonferroni corrected p-value	t	Mean difference 95% confidence interval
step 1 Vs. step 2	.005	**.011**	-2.820	-3.920 ; -0.693
step 1 Vs step 3	.77	.77	0.292	-1.299 ; 1.749
step 1 Vs step 4	.020	**.029**	2.340	0.281 ; 3.302
step 1 Vs step 5	.006	.016	2.756	0.621 ; 3.753
step 2 Vs step 3	.001	**.004**	3.294	1.015 ; 4.048
step 2 Vs step 4	< .001	**<.001**	5.379	2.595 ; 5.601
step 2 Vs step 5	< .001	**<.001**	5.689	2.935 ; 6.052
step 3 Vs step 4	.029	**.041**	2.198	0.161 ; 2.972
step 3 Vs step 5	.009	**.015**	2.643	0.497 ; 3.427
step 4 Vs step 5	.59	.66	0.538	-1.056 ; 1.847

Supplementary Figure 2. Step-by-step distribution of within subject average rating and standard deviation . *Both panels represent the standard deviation of the ratings (y-axis), depending either on their mean rating (left panel) or their number of steps (right panel). On the left panel, for each subject there are 5 dots in the cloud, corresponding to each of the 5 steps. The dots are color coded as in the right panel. The cloud plot shows that the colors (steps) are not randomly distributed along the mean rating but rather form a relatively ordered gradient of step related colors along the x-axis (mean rating). The right panel shows the distribution of this standard deviation for each step and the result of pair-wise t-test with Bonferroni correction.*

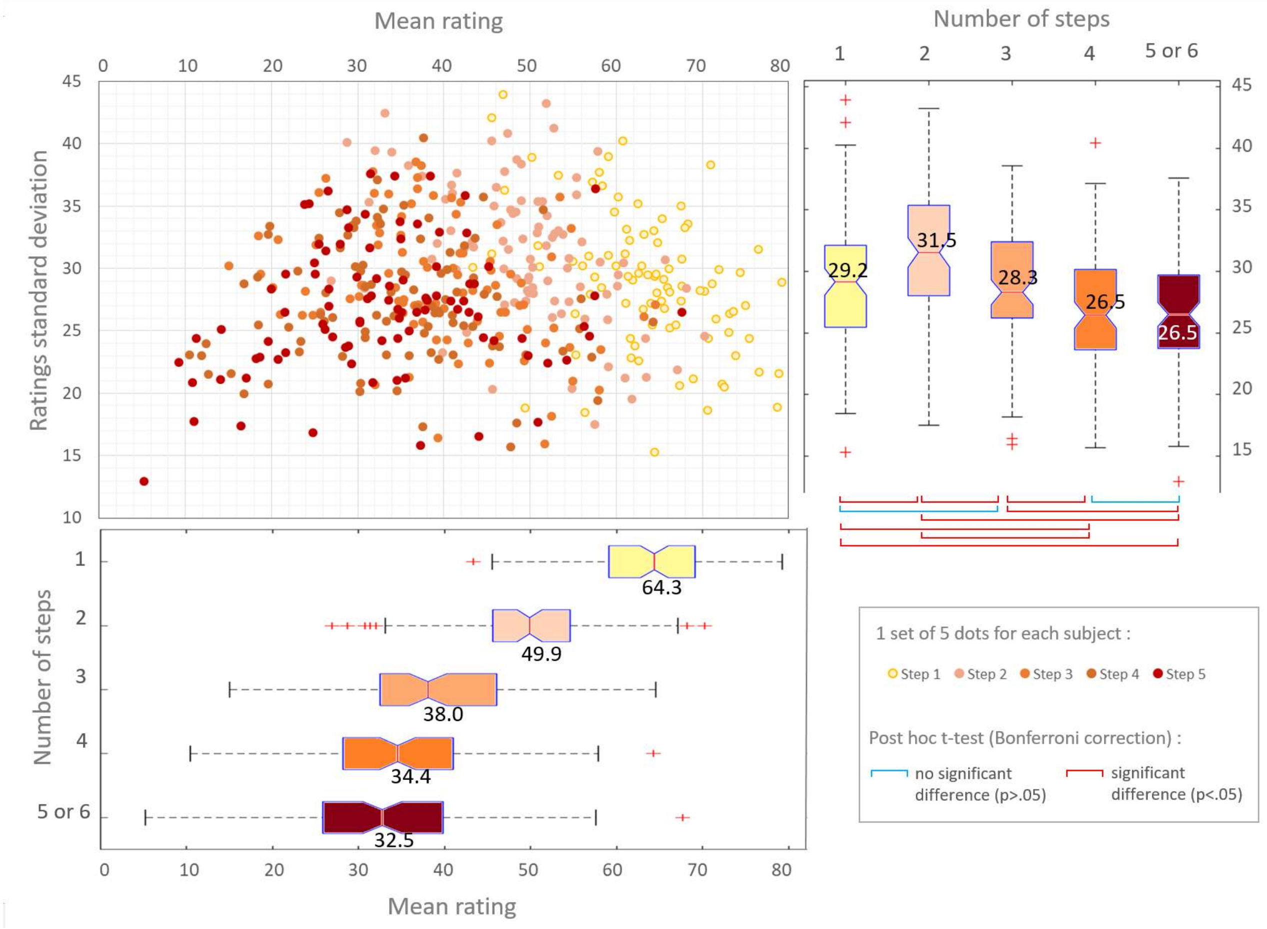

PART4 - FUNCTIONAL MRI

Supplementary Results 1: Brain activation associated with semantic relatedness judgments as a function of theoretical semantic distance

Step 1: The main clusters of positive parametric association were located in the left precentral gyrus (close to the hand region) and in the right calcarine and lingual gyri. Smaller clusters were present in the bilateral middle occipital gyri, the left superior occipital and superior parietal gyri, and in the cerebellum. Besides the left precentral cluster, the other clusters overlap with those previously described in the main positive map above.

Step 2: Compared to the map of step 1, the clusters in the right calcarine and lingual gyri were still present and remained of similar size, additional and more extended clusters appeared in parieto-occipital regions. The cluster in the precentral gyrus was smaller. Additional smaller clusters were observed in the left opercular and orbital parts of the IFG and the superior frontal gyrus and in the left insula.

Step 3: Compared to the map of 2 steps, the occipital and parietal clusters remained (in the right calcarine and lingual gyri, the bilateral inferior parietal cortex with a left predominance) but were less expanded, while the clusters in the left inferior frontal, insula and lateral temporal lobe were larger. The main cluster was located in the left IFG, with a peak in the orbital part, extending in the anterior insula. Smaller frontal clusters were located in the left SMA, the left medial superior frontal gyrus, the left precentral gyrus and the left superior frontal gyrus. In the left temporal lobe appeared two clusters in the pMTG and the pITG.

Step 4: The map for step 4 shows little differences from the previous map of step 3, with a few more significant voxels in the left IFG, the left medial superior frontal gyrus and the left pMTG. On the opposite, the parietal and occipital clusters, as well as the left pITG cluster, are less present than for trials of 3 steps.

Step 5 and 6: For trials with the most distant steps, only six clusters remained significant, located in the right calcarine region, the left middle occipital and inferior parietal regions, and the triangular part of the left IFG. In each of the five theoretical distance group, there was also a positive parametric effect in the cerebellum.

Supplementary Table 4. Whole brain peaks associated with the positive (A) and negative (B) parametric effect of rating

A. Positive parametric effect of rating

Region (aal)	Lat.	Peak (MNI, mm)			T_{peak}	P_{voxel} (FWE)	k
		x	y	z	T		
Cerebellum	R	27.5	-68.5	-29.5	13.708	<.001	334
Calcarine G.	R	10	-86	0.5	12.952	<.001	114
Cerebellum	R	35	-68.5	-42	12.316	<.001	150
Intraparietal S.	L	-40	-43.5	43	12.204	<.001	531
Anterio insula G.	L	-32.5	24	-4.5	11.970	<.001	760
Cerebellum	R	15	-58.5	-47	11.842	<.001	44
Caudate N.	L	-10	16.5	3	11.455	<.001	127
Cingulum mid G.	L	-2.5	9	28	11.303	<.001	25
Frontal sup medial G.	L	-5	31.5	40.5	9.725	<.001	40
Thalamus N.	L	-5	-23.5	0.5	9.385	<.001	9
Thalamus N.	L	-10	-16	8	9.369	<.001	35

Temporal inf G.	L	-55	-43.5	-14.5	9.251	<.001	33
Caudate N.	R	12.5	16.5	3	9.167	<.001	54
Intraparietal S.	R	32.5	-68.5	45.5	8.769	<.001	333
Sup frontal S.	L	-22.5	1.5	55.5	8.567	<.001	91
Temporal mid G.	L	-65	-41	-4.5	8.182	<.001	105
Supp motor area G.	L	-2.5	16.5	60.5	7.979	<.001	15
Cerebellum	L	-5	-76	-27	7.909	<.001	18
Temporal inf G.	R	55	-48.5	-12	7.711	<.001	24
Temporal mid G.	R	42.5	-66	5.5	7.604	<.001	21
Occipital lat G.	L	-20	-98.5	5.5	7.541	<.001	17
Calcarine G.	L	-12.5	-96	-2	7.431	<.001	8
Cingulum post G.	L	-2.5	-33.5	33	7.374	<.001	5
Insula anterior G.	R	37.5	21.5	-2	7.297	<.001	26
Supp motor area G.	L	-12.5	14	63	7.257	<.001	31
Cingulum mid G.	R	5	6.5	28	6.895	<.001	9
Occipital lat G.	L	-30	-91	10.5	6.848	<.001	6
Frontal inf oper	R	45	9	20.5	6.804	<.001	23
Cerebellum	L	-25	-68.5	-29.5	6.558	<.001	7
Frontal sup G.	L	-12.5	36.5	50.5	6.080	.001	8
Occipital sup G.	R	20	-83.5	23	6.046	.001	7
Frontal sup G.	L	-10	46.5	43	6.023	.001	16

B. Negative parametric effect of rating

Region (aal)	Lat.	Peak (MNI, mm)			T_{peak}	P_{voxel} (FWE)	k
		x	y	z	T		
Calcarine G.	L	-7.5	-86	0.5	11.168	<.001	21
Insula anterior G.	L	-40	-8.5	-12	8.622	<.001	46
Lingual G.	L	-20	-53.5	-7	8.502	<.001	8
Occipital sup G.	L	-15	-88.5	25.5	8.396	<.001	31
Lingual G.	L	-10	-81	-7	7.762	<.001	79
Temporal pole sup G.	R	40	-1	-14.5	7.592	<.001	20
Cerebellum	L	-15	-56	-47	7.552	<.001	5
Fusiform G.	L	-30	-8.5	-19.5	7.351	<.001	5
Precuneus G.	R	12.5	-53.5	8	7.173	<.001	24
Calcarine G.	R	15	-93.5	5.5	7.066	<.001	9
Fusiform G.	R	30	-6	-22	6.869	<.001	5
Cingulum mid G.	R	2.5	-26	45.5	6.866	<.001	14
Precuneus G.	R	5	-51	18	6.744	<.001	12
Parahippocampal G.	L	-32.5	-31	-17	6.502	<.001	5
Cuneus G.	L	-7.5	-88.5	20.5	6.498	<.001	9
Precuneus G.	R	5	-51	35.5	6.458	<.001	8
Insula anterior G.	R	35	11.5	10.5	6.273	<.001	7
Sup frontal S.	R	22.5	31.5	33	6.255	<.001	8
Sup frontal S.	R	27.5	36.5	43	6.095	.001	8
Temporal sup G.	L	-45	-28.5	5.5	6.038	.001	10

Cuneus G.	L	-2.5	-81	25.5	6.031	.001	5
Supramarginal G.	R	57.5	-48.5	30.5	5.882	.002	11
Sup frontal S.	R	27.5	56.5	15.5	5.547	.007	5

Supplementary Table 5. Parametric effect of rating for different theoretical distances

A. Parametric effect of rating for word pairs of step 1

Region (aal)	Lat.	Peak (MNI, mm)			Tpeak	Pvoxel (FWE)	k
		x	y	z	T		
Positive parametric effect							
Calcarine G.	R	10	-83.5	0.5	10.885	<.001	52
Precentral G.	L	-32.5	-26	60.5	7.824	<.001	87
Occipital mid G.	L	-30	-86	18	6.866	<.001	7
Occipital mid G.	L	-27.5	-71	25.5	6.813	<.001	11
Cerebellum	R	22.5	-53.5	-22	6.645	<.001	14
Cerebellum	R	12.5	-58.5	-49.5	6.43	<.001	7
Parietal sup G.	L	-17.5	-66	45.5	6.196	<.001	10
Occipital mid G.	R	30	-71	23	6.1	.001	11
Occipital sup G.	L	-22.5	-71	40.5	6.035	.001	5
Occipital mid G.	L	-20	-98.5	5.5	6.013	.001	6
Cerebellum	R	5	-61	-17	5.718	.004	5
Negative parametric effect							
Calcarine G.	L	-7.5	-86	-2	8.211	<.001	16

B. Parametric effect of rating for word pairs of step 2

Region (aal)	Lat.	Peak (MNI, mm)			Tpeak	Pvoxel (FWE)	k
		x	y	z	T		
Positive parametric effect							
Calcarine G.	R	10	-86	0.5	11.628	<.001	39
Cerebellum	R	12.5	-58.5	-49.5	9.488	<.001	12
Occipital sup G.	L	-25	-73.5	40.5	9.075	<.001	137
Parietal inf G.	L	-40	-43.5	45.5	8.993	<.001	174
Frontal inf orb G.	L	-42.5	46.5	-9.5	8.884	<.001	26
Lingual G.	R	12.5	-73.5	-7	8.482	<.001	37
Cerebellum	R	35	-68.5	-27	7.546	<.001	38
Occipital mid G.	R	30	-76	30.5	7.354	<.001	77
Cerebellum	R	12.5	-78.5	-22	6.982	<.001	29
Occipital mid G.	L	-20	-98.5	5.5	6.947	<.001	15
Frontal sup G.	L	-22.5	4	55.5	6.923	<.001	25
Supramarginal G.	R	42.5	-38.5	43	6.871	<.001	26
Postcentral G.	L	-37.5	-21	50.5	6.869	<.001	53
Insula	L	-35	19	3	6.765	<.001	31
Frontal inf oper G.	L	-47.5	9	20.5	6.754	<.001	26
Cerebellum	R	35	-68.5	-42	6.025	.001	17

Frontal inf oper G.	L	-55	14	8	5.989	.001	6
Precentral G.	L	-45	1.5	35.5	5.706	.004	9
Negative parametric effect							
Calcarine G.	L	-7.5	-86	0.5	10.511	<.001	17
Lingual G.	L	-15	-71	-7	8.513	<.001	39
Lingual G.	L	-10	-81	-7	8.268	<.001	
Occipital sup G.	L	-17.5	-88.5	23	6.442	<.001	9

C. <u>Parametric effect of rating for word pairs of step 3</u>

Region (aal)	Lat.	Peak (MNI, mm)			Tpeak	Pvoxel (FWE)	k
		x	y	z	T		
Positive parametric effect							
Calcarine G.	R	10	-86	0.5	11.984	<.001	61
Parietal inf G.	L	-32.5	-58.5	40.5	10.314	<.001	306
Cerebellum	R	27.5	-66	-32	9.992	<.001	78
Cerebellum	R	10	-78.5	-29.5	9.669	<.001	58
Frontal inf orb	L	-45	41.5	-4.5	9.246	<.001	277
Cerebellum	R	32.5	-71	-42	8.962	<.001	79
Frontal sup medial G.	L	-5	31.5	40.5	8.961	<.001	20
Cerebellum	R	12.5	-58.5	-49.5	8.775	<.001	14
Caudate N.	L	-12.5	14	13	8.378	<.001	7
Precentral G.	L	-45	9	45.5	7.915	<.001	60
Supp motor area	L	-2.5	19	60.5	7.609	<.001	7
Temporal mid G.	L	-65	-38.5	-4.5	7.382	<.001	73
Angular G.	R	32.5	-68.5	45.5	7.198	<.001	11
Frontal mid G.	L	-25	6.5	53	7.142	<.001	34
Supp motor area	L	-12.5	21.5	60.5	6.776	<.001	17
Temporal inf G.	L	-55	-41	-12	6.651	<.001	14
Temporal inf G.	R	57.5	-48.5	-14.5	6.475	<.001	6
Parietal inf G.	R	37.5	-41	38	6.152	0.001	16
Frontal sup G.	L	-15	41.5	45.5	5.893	0.002	6
Cingulum ant G.	L	-2.5	6.5	28	5.797	0.003	5
Negative parametric effect							
Calcarine G.	L	-7.5	-86	0.5	10.103	<.001	19
Lingual G.	L	-12.5	-73.5	-4.5	7.599	<.001	41
Calcarine G.	R	15	-93.5	5.5	6.904	<.001	8
Occipital mid G.	L	-17.5	-88.5	20.5	6.582	<.001	17

D. Parametric effect of rating for word pairs of step 4

Region (aal)	Lat.	Peak (MNI, mm)			Tpeak	Pvoxel (FWE)	k
		x	y	z	T		
Positive parametric effect							
Calcarine G.	R	10	-86	0.5	10.889	<.001	24
Parietal inf G.	L	-42.5	-46	50.5	9.407	<.001	201
Supp motor area	L	-2.5	16.5	60.5	9.194	<.001	18
Frontal inf oper G.	L	-55	19	15.5	8.949	<.001	396
Frontal sup medial G.	L	-2.5	34	45.5	8.752	<.001	22
Cerebellum	R	10	-81	-29.5	8.294	<.001	90
Frontal mid G.	L	-37.5	6.5	53	7.85	<.001	38
Temporal mid	L	-47.5	-33.5	-2	7.584	<.001	92
Lingual G.	R	12.5	-81	-12	7.579	<.001	18
Caudate N.	L	-12.5	19	5.5	7.478	<.001	11
Supp motor area	L	-12.5	11.5	63	7.444	<.001	10
Frontal sup G.	L	-12.5	21.5	58	7.337	<.001	30
Cerebellum	R	22.5	-78.5	-47	6.878	<.001	6
Frontal sup medial G.	L	-7.5	46.5	45.5	6.436	<.001	5
Frontal mid G.	L	-22.5	4	53	6.397	<.001	17
Temporal inf G.	L	-57.5	-43.5	-14.5	6.341	<.001	8
Negative parametric effect							
Calcarine G.	L	-7.5	-86	-2	9.5	<.001	17
Lingual G.	L	-10	-78.5	-7	7.116	<.001	14
Occipital sup G.	L	-15	-86	20.5	6.592	<.001	17
Calcarine G.	R	15	-96	5.5	6.461	<.001	10

E. Parametric effect of rating for word pairs of step 5 and 6

Region (aal)	Lat.	Peak (MNI, mm)			Tpeak	Pvoxel (FWE)	k
		x	y	z	T		
Positive parametric effect							
Calcarine G.	R	10	-86	0.5	7.473	<.001	14
Cerebellum	R	25	-68.5	-29.5	6.709	<.001	5
Occipital mid G.	L	-27.5	-71	35.5	6.451	<.001	12
Frontal inf tri G.	L	-52.5	19	5.5	6.434	<.001	16
Cerebellum	R	37.5	-68.5	-42	6.047	.001	5
Parietal inf G.	L	-45	-43.5	50.5	6.038	.001	5
Cerebellum	R	10	-78.5	-27	6.035	.001	5
Parietal inf G.	L	-45	-38.5	45.5	5.847	.002	8
Parietal inf G.	L	-52.5	-41	50.5	5.669	.004	

Negative parametric effect

Calcarine G.	L	-7.5	-86	0.5	9.6	<.001	15
Lingual G.	L	-12.5	-78.5	-4.5	6.749	<.001	13
Cuneus G.	L	-12.5	-88.5	25.5	6.61	<.001	11

PART 5 - BRAIN NETWORK CONTRIBUTIONS ACROSS STEPS OF THEORETICAL SEMANTIC DISTANCE

Supplementary Table 6. Statistics on the beta regressor "RJT reflection period * RJT rating" in the overlap of the rating-modulated map with the functional networks [5]. *Step-by-step : mean values and correlation with the number of steps. Values in bold font are those with a significant p-value after Bonferroni correction.*

	Mean of the beta regressor "RJT reflection period * RJT rating" over all runs and all subjects					Kendall correlation of the beta regressor with the steps		
	Step 1	Step 2	Step 3	Step 4	Step 5	tau	*p* uncorrected	*p* Bonferroni
Regions whose activity increases with the rating								
**N1 visual**	**0.007**	**0.006**	**0.004**	**0.004**	**0.003**	**-0.161**	**< .001**	**< .001**
**N2 visual**	**0.017**	**0.014**	**0.010**	**0.009**	**0.007**	**-0.230**	**< .001**	**< .001**
N5 attentional	0.006	0.006	0.005	0.004	0.004	-0.095	.005	.086
N6 attentional	0.004	0.004	0.003	0.003	0.004	-0.053	.119	>1
N8 salience	0.004	0.004	0.004	0.005	0.005	0.0353	.299	>1
N11 control	0.005	0.005	0.004	0.004	0.003	-0.063	.063	>1
N12 control	0.004	0.005	0.005	0.005	0.005	0.043	.205	>1
N13 control	0.004	0.004	0.005	0.005	0.006	0.098	.004	.067
**N14 default**	**0.001**	**0.003**	**0.006**	**0.007**	**0.004**	**0.166**	**< .001**	**< .001**
N16 default	0.005	0.004	0.005	0.004	0.005	0.029	.399	>1
**N17 default**	**0.003**	**0.004**	**0.006**	**0.007**	**0.006**	**0.242**	**< .001**	**< .001**
Regions whose activity decreases with the rating								
N1 visual	-0.006	-0.006	-0.006	-0.007	-0.009	-0.068	.046	.784
N2 visual	-0.006	-0.006	-0.006	-0.006	-0.008	-0.065	.056	.943
N4 motor	-0.001	-0.004	-0.004	-0.003	-0.005	-0.060	.078	>1
N7 attentional	-0.002	-0.004	-0.005	-0.005	-0.005	-0.094	.006	.100
N15 default	-0.005	-0.004	-0.003	-0.003	-0.003	0.084	.084	>1
N16 default	-0.003	-0.004	-0.003	-0.004	-0.004	-0.038	.267	>1

Supplementary Table 7. Statistics on the beta regressor "RJT reflection period * RJT rating" in the SCN and MDN. *Step-by-step: mean values and correlation with the number of steps. Values in bold font are those with a significant p-value after Bonferroni correction.*

	Mean of the beta regressor "RJT reflection period * RJT rating" over all runs and all subjects					Kendall correlation of the beta regressor with the steps		
	Step 1	Step 2	Step 3	Step 4	Step 5	tau	*p*	
							uncorrected	Bonferroni
SCN specific	1.28E-3	2.21E-3	3.70E-3	4.28E-3	3.75E-3	**0.217**	**< .001**	**< .001**
MDN specific	2.22E-3	1.87E-3	1.25E-3	1.03E-3	8.35E-4	**-0.121**	**< .001**	**.0011**
Shared by MDN and SCN	1.57E-3	2.81E-3	3.08E-3	3.99E-3	3.95E-3	**0.131**	**< .001**	**< .001**

PART 6 - STEP-BY-STEP ANALYSIS AFTER DOWN-SAMPLING THE NUMBER OF TRIALS FOR EACH STEP

Supplementary Analysis 3: Step-by-step analysis after down-sampling the number of trials for each step

To make sure that the results observed in the step-by-step analysis were not affected by a difference of power between the different step category, we performed a downsampling of the trials of 2, 3 and 4 steps to 105 trials. Thus, each distance condition (step 1 to 5/6) has the same number of trials.

Method

We repeated the fMRI data analysis as described in the paper, but this time with a downsampling for the trials of 2, 3 and 4 steps. When creating the GLM for each subject at the first-level analysis, only 105 trials were modeled for those steps. These 105 trials were randomly selected among all the trials of the step, using the "randperm" function in Matlab. This random selection of 105 trials for each step was newly performed for each of the 93 subject. The subsequent steps of the analysis remained the same as described in the method of the paper.

Results

The result of this step-by-step analysis after the downsampling are displayed on Supplementary Figure 3, and the overlaps of these maps with the networks of Yeo [5] on Supplementary Figure 4. Overall, we observed the same tendency as in the main analysis (without downsampling).
The results of the extraction of the parametric beta regressor obtained with this downsampled analysis, in each regions of the main map parceled according to the 17 functional networks of Yeo et al. [5] are displayed on Supplementary Figure 5 and Supplementary Table 8. Again, we observed similar results as in the main analysis (without the downsampling) : the regressor significantly increased with the number of steps in the regions belonging to the default subnetworks N14 and N17, and significantly decreased in the regions overlapping with the visual networks N1 and N2.

The overlap of the maps resulting from the downsampled analysis with the SCN and the MDN are displayed on Supplementary Figure 6A, and the results of the extraction of the beta regressor in those networks are shown in Supplementary Figure 6B and Supplementary Table 9. Overall, we observed similar results as in the main analysis (without the downsampling), in particular the parametric beta regressor significantly increased with the number of steps in the SCN regions (both in the SCN specific regions and those belonging to both the SCN and the MDN) and it significantly decreased in the MDN specific regions.

Supplementary Figure 3. Step-by-step analysis result after the downsampling. *Positive (red) and negative (blue) parametric effect of the rating on the reflection period for different theoretical distances.*

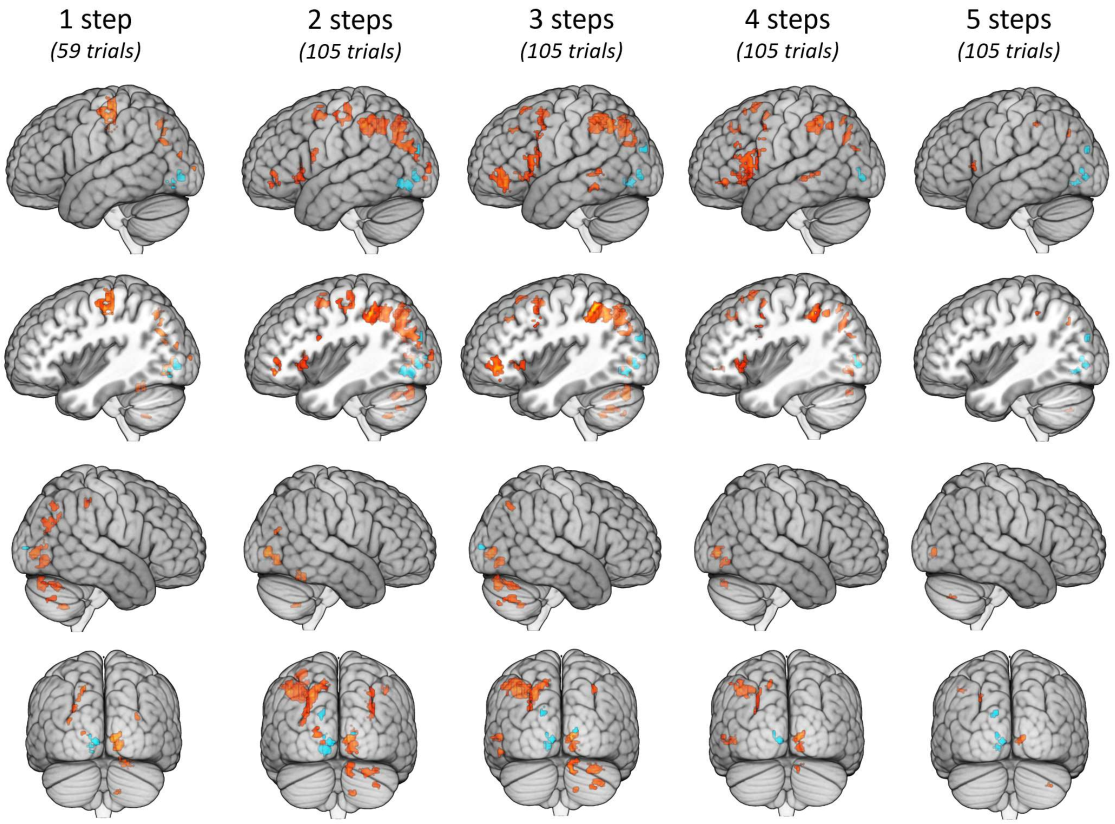

Supplementary Figure 4. Number of voxels in the step-by-step maps (downsampled analysis) of the positive parametric effect of the ratings overlapping with the functional networks [5]. *The height of each tower reflects the number of voxels in the map. For clarity purpose, the different subnetworks were merged by functional role. To better estimate the margin of error of this quantification due to the voxels located in the paracortical white matter, we isolated them from those belonging to the cerebellum or the basal nuclei.*

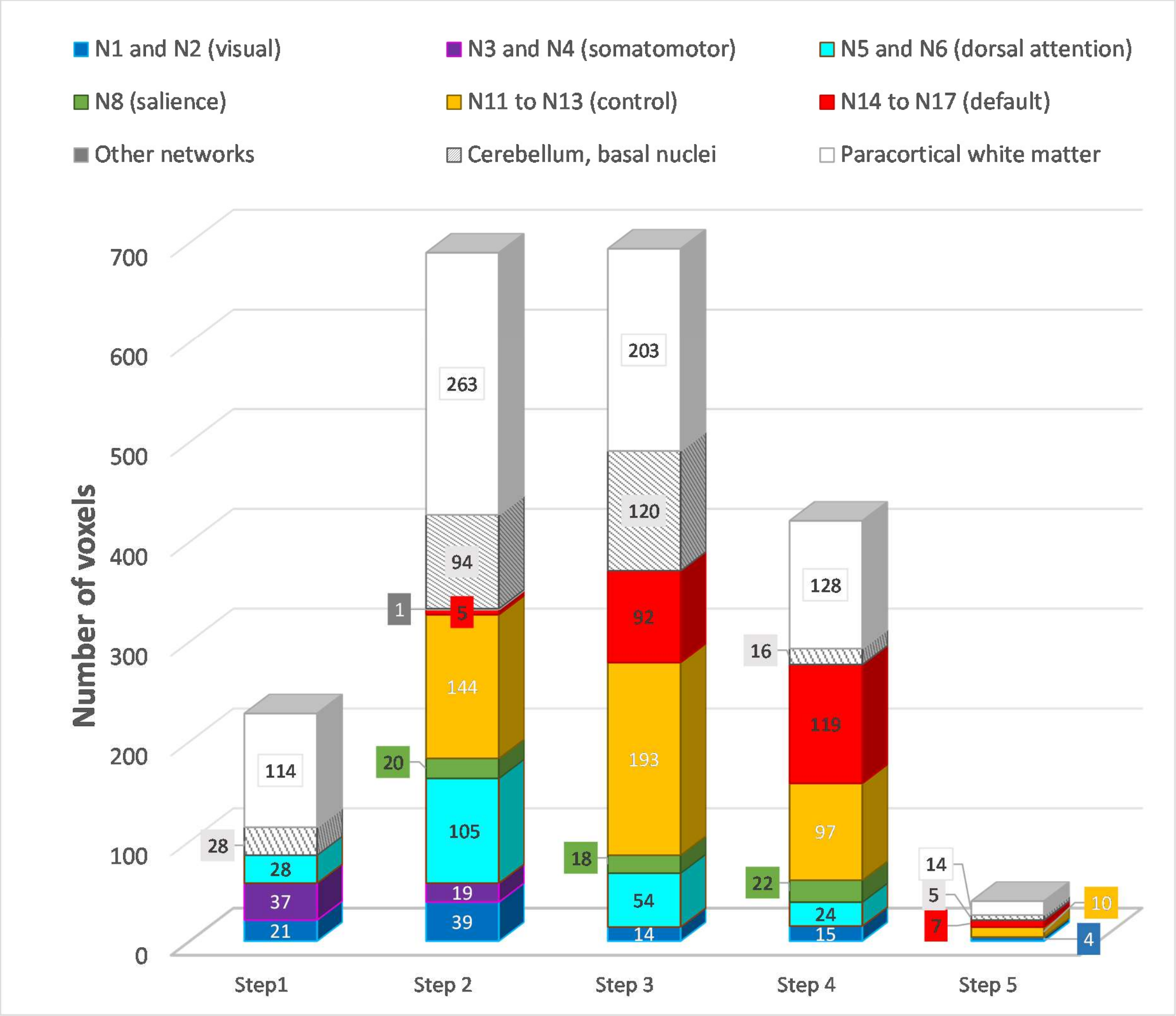

Supplementary Figure 5. Average value of the parametric regressor "RJT reflection period * RJT rating" (downsampled analysis) in rating-modulated regions depending on the theoretical distances (steps) in clusters whose activity increases (A) and decreases (B) with the ratings. *The dotted lines represent overlaps of less than 100 voxels; the solid lines represent overlaps of more than 100 voxels. Kendall correlations with Bonferroni corrected p-values: * < .05 ; **< .01 ; *** < .001*

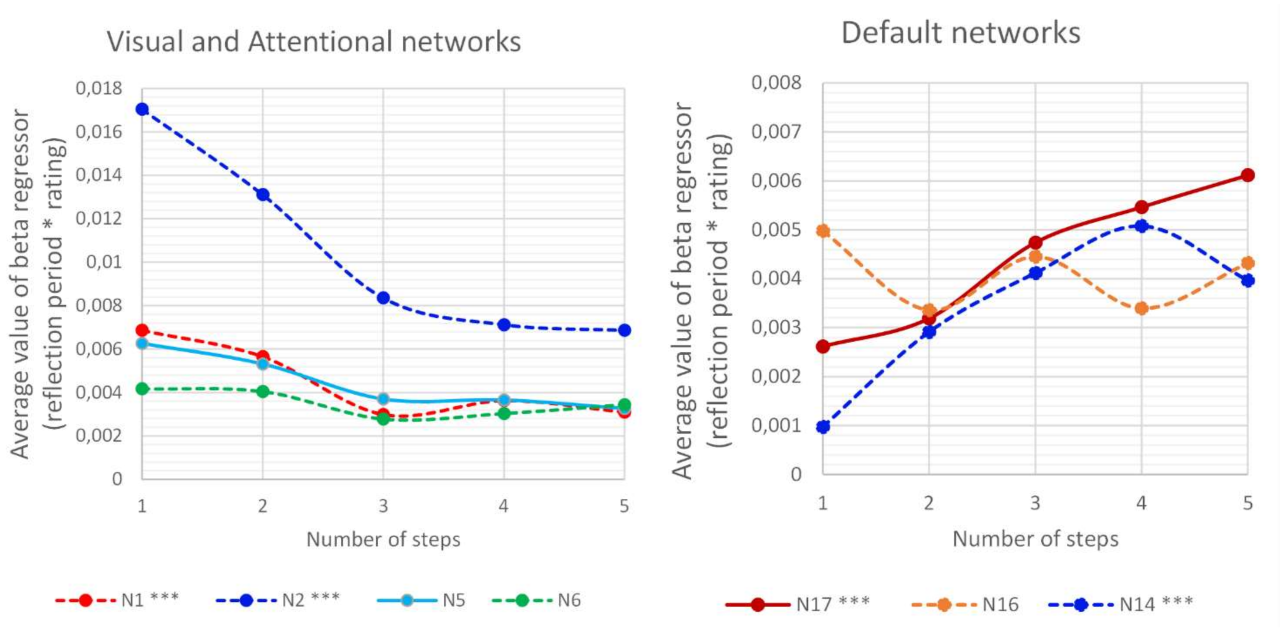

Supplementary Table 8. Statistics on the beta regressor "RJT reflection period * RJT rating" in the overlap of the rating sensitive regions with the functional networks [5] . *Step-by-step, after downsampling to 105 trials for each step (except for step 1 : 59 trials) : mean values of the beta regressor and correlation with the number of steps. Values in bold font are those with a significant p-value after Bonferroni correction.*

	Mean of the beta regressor "RJT reflection period * RJT rating" over all runs and all subjects					Kendall correlation of the beta regressor with the steps		
	Step 1	Step 2	Step 3	Step 4	Step 5	tau	*p*	
							uncorrected	Bonferroni
Regions whose activity increases with the rating								
**N1 visual**	**6.9E-3**	**5.7E-3**	**3.0E-3**	**3.7E-3**	**3.1E-3**	**-0.15**	**< .001**	**< .001**
**N2 visual**	**1.7E-2**	**1.3E-2**	**8.4E-3**	**7.1E-3**	**6.9E-3**	**-0.24**	**< .001**	**< .001**
N5 attentional	6.3E-3	5.3E-3	3.7E-3	3.7E-3	3.3E-3	-0.095	.0050	.065
N6 attentional	4.2E-3	4.1E-3	2.8E-3	3.0E-3	3.5E-3	-0.061	.071	.93
N8 salience	3.5E-3	4.0E-3	3.7E-3	4.5E-3	4.5E-3	0.029	.39	>1
N11 control	5.0E-3	4.7E-3	3.6E-3	3.7E-3	3.2E-3	-0.062	.056	.73
N12 control	3.8E-3	4.7E-3	4.1E-3	4.3E-3	4.7E-3	0.043	.20	>1
N13 control	3.7E-3	4.1E-3	4.9E-3	4.1E-3	5.6E-3	0.081	.016	.21
**N14 default**	**9.7E-4**	**2.9E-3**	**4.1E-3**	**5.1E-3**	**4.0E-3**	**0.17**	**< .001**	**< .001**
N16 default	5.0E-3	3.4E-3	4.5E-3	3.4E-3	4.3E-3	0.010	.76	>1
**N17 default**	**2.6E-3**	**3.2E-3**	**4.8E-3**	**5.5E-3**	**6.1E-3**	**0.27**	**< .001**	**< .001**

Supplementary Figure 6. Exploring RJT brain correlates in regard to the semantic cognition related networks : downsampled analysis. *(A) Number of voxels in the step-by-step maps of positive parametric effect of the rating overlapping with the MDN and the SCN. The height of each tower reflects the number of voxels in the map. To better estimate the amount of voxels potentially belonging to other networks than the SCN or the MDN, we isolated those voxels ("remaining voxels") from those belonging to the cerebellum or basal nuclei. (B) Average value of the beta regressor "reflection period * rating" per step in the SCN and the MDN. The dotted lines represent overlaps of less than 100 voxels; the solid lines represent overlaps of more than 100 voxels. Bonferroni corrected p-values (Kendall correlation): ** <.01; ***<.001*

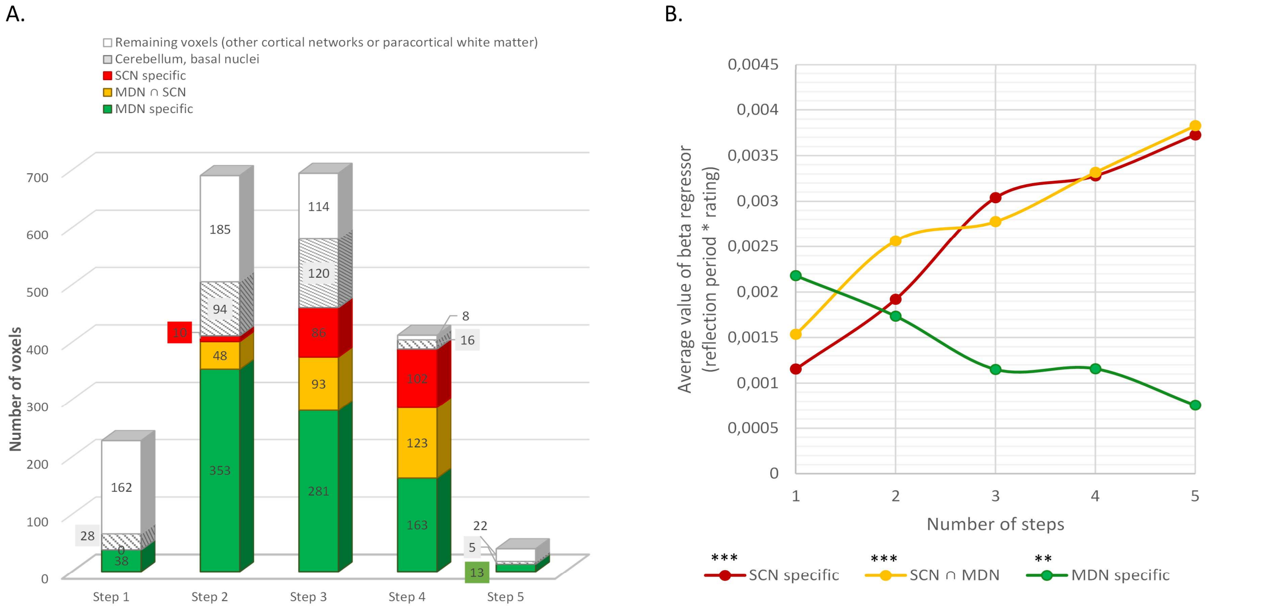

Supplementary Table 9. Statistics on the beta regressor "RJT reflection period * RJT rating" in the SCN and MDN. *Step-by-step, after downsampling to 105 trials for each step (except for step 1: 59 trials) : mean values and correlation with the number of steps*

	Mean of the beta regressor "RJT reflection period * RJT rating" over all runs and all subjects					Kendall correlation of the beta regressor with the steps		
	Step 1	Step 2	Step 3	Step 4	Step 5	tau	*p*	
							uncorrected	Bonferroni
SCN specific	1.2E-3	1.9E-3	3.0E-3	3.3E-3	3.7E-3	0.21	< .001	< .001
MDN specific	2.2E-3	1.7E-3	1.2E-3	1.2E-3	7.6E-4	-0.10	.0021	.0063
Shared by MDN and SCN	1.5E-3	2.6E-3	2.8E-3	3.3E-3	3.8E-3	0.13	< .001	< .001

SUPPLEMENTARY REFERENCES

1. Bernard, M., Kenett, Y., Ovando-Tellez, M., Benedek, M. & Volle, E. Building Individual Semantic Networks and Exploring Their Relationships with Creativity. (2019).
2. Benedek, M. et al. How semantic memory structure and intelligence contribute to creative thought: a network science approach. Think. Reason. 23, 158–183 (2017).
3. Ovando-Tellez, M. et al. Brain connectivity–based prediction of real-life creativity is mediated by semantic memory structure. Sci. Adv. 8, eabl4294 (2022).
4. He, L. et al. The relation between semantic memory structure, associative abilities, and verbal and figural creativity. Think. Reason. 27, 268–293 (2021).
5. Yeo, B. T. T. et al. The organization of the human cerebral cortex estimated by intrinsic functional connectivity. J. Neurophysiol. 106, 1125–1165 (2011).
