# Supplementary material for: Creative connections: the neural correlates of semantic relatedness are associated with creativity
Source: Commun Biol. 2024 Jul 4;7:810. doi: 10.1038/s42003-024-06493-y (PMC11222432; doi:10.1038/s42003-024-06493-y)
Supplement: Supplementary file 3 — ReportingSummary [file 42003_2024_6493_MOESM3_ESM.pdf]

Corresponding author(s): Caroline Herault and Emmanuelle Volle
Last updated by author(s): 06/20/2024

Reporting Summary

Nature Portfolio wishes to improve the reproducibility of the work that we publish. This form provides structure for consistency and transparency in reporting. For further information on Nature Portfolio policies, see our Editorial Policies and the Editorial Policy Checklist.

Statistics

For all statistical analyses, confirm that the following items are present in the figure legend, table legend, main text, or Methods section.

n/a	Confirmed	
☐	☒	The exact sample size (n) for each experimental group/condition, given as a discrete number and unit of measurement
☐	☒	A statement on whether measurements were taken from distinct samples or whether the same sample was measured repeatedly
☐	☒	The statistical test(s) used AND whether they are one- or two-sided<br>*Only common tests should be described solely by name; describe more complex techniques in the Methods section.*
☐	☒	A description of all covariates tested
☐	☒	A description of any assumptions or corrections, such as tests of normality and adjustment for multiple comparisons
☐	☒	A full description of the statistical parameters including central tendency (e.g. means) or other basic estimates (e.g. regression coefficient) AND variation (e.g. standard deviation) or associated estimates of uncertainty (e.g. confidence intervals)
☐	☒	For null hypothesis testing, the test statistic (e.g. F, t, r) with confidence intervals, effect sizes, degrees of freedom and P value noted<br>*Give P values as exact values whenever suitable.*
☒	☐	For Bayesian analysis, information on the choice of priors and Markov chain Monte Carlo settings
☒	☐	For hierarchical and complex designs, identification of the appropriate level for tests and full reporting of outcomes
☐	☒	Estimates of effect sizes (e.g. Cohen's d, Pearson's r), indicating how they were calculated

Our web collection on statistics for biologists contains articles on many of the points above.

Software and code

Policy information about availability of computer code

Data collection	Behavioral data were collected using custom scripts running on Matlab (Matlab R2017b, The MathWorks, Inc.,USA)
Data analysis	The analyses were conducted using MATLAB and Python, with open toolboxes available online as described in Materials and Methods (SPM: www.fil.ion.ucl.ac.uk/spm/software/spm12/ ; Nilearn: https://nilearn.github.io/stable/index.html ; Nibabel : https://nipy.org/nibabel/ ; TEDANA: https://tedana.readthedocs.io/en/stable/ ;).

For manuscripts utilizing custom algorithms or software that are central to the research but not yet described in published literature, software must be made available to editors and reviewers. We strongly encourage code deposition in a community repository (e.g. GitHub). See the Nature Portfolio guidelines for submitting code & software for further information.

Data

Policy information about availability of data

All manuscripts must include a data availability statement. This statement should provide the following information, where applicable:

- Accession codes, unique identifiers, or web links for publicly available datasets
- A description of any restrictions on data availability
- For clinical datasets or third party data, please ensure that the statement adheres to our policy

The data needed to evaluate the conclusions in the paper are available on Dryad (https://datadryad.org/stash/share/TKTOZFGVfYXLItnLDvOL4C2-

RhlNbBZqyvzTCSoxwN4). The maps presented in the results are available on Neurovault (https://neurovault.org/collections/VMEBGFOG/).
The code used for the analysis of the data is available at https://github.com/CaroHerault/CreativeConnections_scripts.

Research involving human participants, their data, or biological material

Policy information about studies with human participants or human data. See also policy information about sex, gender (identity/presentation), and sexual orientation and race, ethnicity and racism.

Reporting on sex and gender	The findings of this study apply to both male and females.<br>Among the 93 participants, 44 were females and 49 males.<br>This sex determination was based on self-report.<br>This information was collected to characterize the population studied, but it was not used in the analysis, as we did not have any hypotheses on sex or gender differences in our effect of interest.
Reporting on race, ethnicity, or other socially relevant groupings	Besides sex and age, no other social characteristic was collected.
Population characteristics	The population characteristics are reported in the Material and Method section of the manuscript.<br>One hundred and one healthy participants were recruited via the RISC platform (https://www.risc.cnrs.fr), all French native speakers, right-handed, with normal or corrected to-normal vision. They all declared no history of neurological or psychiatric disease, no evolutive neuropsychiatric condition, no psychotropic medication, no drug abuse or cognitive difficulties. Eight participants were excluded from the fMRI analysis: six participants were excluded because of the discovery of brain abnormalities, one participant fell asleep during the acquisition, and another participant had a claustrophobia episode at the beginning of the scanning.<br>The final sample was hence composed of 93 participants (Mean age = 25.4 years; SD = 3.4 years; 44 women).
Recruitment	One hundred and one healthy participants were recruited via the RISC platform (https://www.risc.cnrs.fr), all French native speakers, right-handed, with normal or corrected to-normal vision. They all declared no history of neurological or psychiatric disease, no evolutive neuropsychiatric condition, no psychotropic medication, no drug abuse or cognitive difficulties. Eight participants were excluded from the fMRI analysis: six participants were excluded because of the discovery of brain abnormalities, one participant fell asleep during the acquisition, and another participant had a claustrophobia episode at the beginning of the scanning.
Ethics oversight	The study was approved by a national ethical committee (CPP Number 180,103; ID-RCB 2017-A03109-44).<br>After being informed of the study, the participants signed a written consent form.

Note that full information on the approval of the study protocol must also be provided in the manuscript.

Field-specific reporting

Please select the one below that is the best fit for your research. If you are not sure, read the appropriate sections before making your selection.

☒ Life sciences ☐ Behavioural & social sciences ☐ Ecological, evolutionary & environmental sciences

For a reference copy of the document with all sections, see nature.com/documents/nr-reporting-summary-flat.pdf

Life sciences study design

All studies must disclose on these points even when the disclosure is negative.

Sample size	This is an exploratory study and effect size was not known.
Data exclusions	Among the 101 subjects recruited, eight participants were excluded from the fMRI analysis: six participants were excluded because of the discovery of brain abnormalities, one participant fell asleep during the acquisition, and another participant had a claustrophobia episode at the beginning of the scanning.<br>The final sample was hence composed of 93 participants
Replication	The results from the analysis were replicated after a downsampling of the trials of each step described in the supplementary material.
Randomization	Randomization was not applicable to our study design because there was only one experimental group.
Blinding	Blinding was not applicable to our study design because there was only one experimental group.

Reporting for specific materials, systems and methods

We require information from authors about some types of materials, experimental systems and methods used in many studies. Here, indicate whether each material, system or method listed is relevant to your study. If you are not sure if a list item applies to your research, read the appropriate section before selecting a response.

Materials & experimental systems

n/a	Involved in the study
☒	☐ Antibodies
☒	☐ Eukaryotic cell lines
☒	☐ Palaeontology and archaeology
☒	☐ Animals and other organisms
☒	☐ Clinical data
☒	☐ Dual use research of concern
☒	☐ Plants

Methods

n/a	Involved in the study
☒	☐ ChIP-seq
☒	☐ Flow cytometry
☐	☒ MRI-based neuroimaging

Magnetic resonance imaging

Experimental design

Design type

Block design

Design specifications

Each of the 595 RJT trials consisted in displaying a word pair on the screen together with a visual scale below it, ranging from 0 (unrelated) to 100 (strongly related). This screen was displayed for four seconds in total divided into a reflection period of two seconds, to ensure a comparable minimum thinking time, and a response period of two seconds. During the first two seconds, the participants studied the word pair but couldn't move the slider yet. Two seconds after stimuli onset, the response period began, the cursor appeared in the middle of the visual scale, and the participants were allowed to move the slider on the visual scale to indicate their rating using a trackball. Participants were instructed to validate their response by clicking the left button of the trackball. The position of the cursor on the scale at the moment of the validation was recorded as the relatedness judgement. The difference of time between the beginning of the response period and the moment of the validation was recorded as the reaction time. When participants did not validate their response, the slider position at the end of the 2-second response period was recorded. After the response period, a blank screen was shown during the inter-trial interval jittered from 0.3 to 0.7 seconds (mean 0.5, steps = 0.05). Task trials were distributed into 6 runs composed of 100 trials each, except for the last run (95 trials). Each run consisted of four blocks of 25 trials each (except the last block of the sixth run with only 20 trials), separated by a 20 second rest period with a cross fixation on the screen. Trials were pseudo-randomly ordered within blocks, such that each block contained a similar proportion of word pairs of each theoretical semantic distance. At the beginning and end of each run, participants had a 10 second rest period with a cross fixation on the screen. During the last two seconds of the fixation cross period, the cross changed color, warning the participant that the task was about to start. Participants had a self-paced break inside the scanner between runs.

Behavioral performance measures

The behevioral measures recorded for each trial were the semantic relatedness rating and the response duration.

Acquisition

Imaging type(s)

functional MRI

Field strength

3T

Sequence & imaging parameters

Six functional runs were acquired during each six task runs using multi-echo echo-planar imaging (EPI) sequences. No dummy scan was recorded during the acquisition. Each run included 335 whole-brain volumes acquired with the following parameters: repetition time (TR) = 1,600 ms, echo times (TE) for echo 1 = 15.2 ms, echo 2 = 37.17 ms and echo 3 = 59.14 ms, flip angle = 73°, 54 slices, slice thickness = 2.50 mm, isotropic voxel size 2.5 mm, Ipat acceleration factor = 2, multi-band = 3 and interleaved slice ordering. After the EPI acquisitions, a T1-weighted structural image was acquired with the following parameters: TR = 2,300 ms, TE = 2.76 ms, flip angle = 9°, 192 sagittal slices with a 1 mm thickness, isotropic voxel size 1 mm, Ipat acceleration factor = 2 and interleaved slice order.

Area of acquisition

Whole brain scan

Diffusion MRI ☐ Used ☒ Not used

Preprocessing

Preprocessing software

The processing of the fMRI data used the afni_proc.py pipeline from the Analysis of Functional Neuroimages software (AFNI; https://afni.nimh.nih.gov). The different preprocessing steps of the data included despiking, slice timing correction and realignment to the first volume (computed on the first echo). The data of each run were preprocessed separately.

Normalization

The data was normalized to the Montreal Neurological Institute (MNI) template brain.

Normalization template

To normalize the data to the MNI template brain, we used using the transformation matrix computed from the normalization of the T1-weighted structural image, performed with the default settings of the computational anatomy toolbox (CAT 12; http://dbm.neuro.uni-jena.de/cat/) implemented in SPM 12.

Noise and artifact removal

The preprocessed fMRI data were denoised using TE-dependent analysis of multi-echo (TEDANA; https://

Noise and artifact removal

tedana.readthedocs.io/en/stable/). The resulting denoised data was co-registered on the T1-weighted structural image using the Statistical Parametric Mapping (SPM) 12 package running in Matlab (Matlab R2017b, The MathWorks, Inc., USA). Furthermore, the resulting denoised and normalized BOLD signal outputted by the TEDANA workflow were analyzed in SPM12 using general linear models (GLM), modeling, besides the conditions of interest (the reflection period, the response period and the cross-fixation period), 27 regressors of no interest to control for effects related to response time for each trial, fatigue across trials, and head motion. These regressors of non-interest included 24 motion regressors (the 6 standard motion parameters during pre-processing, their first temporal derivatives, their values squared and the first temporal derivatives squared), the rank, onset time and reaction time of each trial.

Volume censoring

Whole brain effects were inclusively masked with an explicit gray matter mask estimated based on the SPM12 gray matter tissue map (x > 0.2).

Statistical modeling & inference

Model type and settings

We used general linear models (GLM) modeling 3 conditions : the reflection period, the response period and the cross-fixation period. The semantic relatedness rating was included as a modulation parameter on the reflection period, with the values normalized at the individual level in the model.
We further included 27 regressors of no interest to control for effects related to response time for each trial, fatigue across trials, and head motion (described in the previous section).
Linear contrasts were then used, concatenating the results of each run, to obtain the subject specific estimates for the parametric effect of the semantic relatedness rating.
These subject specific estimates were then entered into a second-level analysis treating subjects as a random effect.

Effect(s) tested

Our analysis focused on the parametric effect of the semantic relatedness rating during the reflection period.

Specify type of analysis: ☐ Whole brain ☐ ROI-based ☒ Both

Anatomical location(s)

In the first part of the study, we ran group analysis involving the whole brain to identify the regions whose activity was varying linearly with the semantic relatedness rating.
In the second part of the study, we ran an inter-individual analysis to test whether the brain activity during the relatedness judgment task (RJT) correlated with the behavioral scores, in particular the individual RJT mean rating and their creativity scores. This second analysis was based on a region of interest (ROI), defined by binarizing the map that resulted of the general positive parametric analysis, used as a mask to extract, for each individual, the beta values in each voxel from the beta nifti files generated by the GLM modeling all trials together. For each subject, we computed the average beta value in this entire map over the six runs. We then computed the Pearson correlation coefficient between this average beta regressor (reflecting the brain activity during the RJT) and each of the six creativity scores as well as the RJT mean rating per subject.

Statistic type for inference

(See Eklund et al. 2016)

Voxel-wise

Correction

The effects were reported when significant at voxel level ($p < .05$, FWE corrected for multiple comparison) and cluster size was ≥ 5 voxels.

Models & analysis

n/a	Involved in the study
☒	☐ Functional and/or effective connectivity
☒	☐ Graph analysis
☒	☐ Multivariate modeling or predictive analysis
